# UBB^+1^ reduces amyloid-β cytotoxicity by activation of autophagy in yeast

**DOI:** 10.18632/aging.203681

**Published:** 2021-11-09

**Authors:** Xin Chen, Ana Joyce Muñoz-Arellano, Dina Petranovic

**Affiliations:** 1 Division of Systems and Synthetic Biology, Department of Biology and Biological Engineering, Chalmers University of Technology, Gothenburg, Sweden; 2 Novo Nordisk Foundation Center for Biosustainability, Chalmers University of Technology, Gothenburg, Sweden

**Keywords:** UBB^+1^, autophagy, amyloid-β, Alzheimer’s disease, yeast

## Abstract

UBB^+1^ is a mutated version of ubiquitin B peptide caused by a transcriptional frameshift due to the RNA polymerase II “slippage”. The accumulation of UBB^+1^ has been linked to ubiquitin-proteasome system (UPS) dysfunction and neurodegeneration. Alzheimer’s disease (AD) is defined as a progressive neurodegeneration and aggregation of amyloid-β peptides (Aβ) is a prominent neuropathological feature of AD. In our previous study, we found that yeast cells expressing UBB^+1^ at lower level display an increased resistance to cellular stresses under conditions of chronological aging. In order to examine the molecular mechanisms behind, here we performed genome-wide transcriptional analyses and molecular/cellular biology assays. We found that low UBB^+1^ expression activated the autophagy pathway, increased vacuolar activity, and promoted transport of autophagic marker ATG8p into vacuole. Furthermore, we introduced low UBB^+1^ expression to our humanized yeast AD models, that constitutively express Aβ42 and Aβ40 peptide, respectively. The co-expression of UBB^+1^ with Aβ42 or Aβ40 peptide led to reduced intracellular Aβ levels, ameliorated viability, and increased chronological life span. In an autophagy deficient background strain (*atg1Δ*), intracellular Aβ levels were not affected by UBB^+1^ expression. Our findings offer insights for reducing intracellular Aβ toxicity via autophagy-dependent cellular pathways under low level of UBB^+1^ expression.

## INTRODUCTION

Proteins are frequently misfolded during the lifetime of a cell, as a consequence of stochastic fluctuations of the structures, genomic mutations, oxidation or other different stress conditions [1]. Misfolded proteins often tend to aggregate due to the exposure of hydrophobic amino acid residues and unstructured polypeptide backbones, which are shielded in a native conformation [2]. Accumulation of misfolded proteins within cellular compartments or tissues is emerging as a major contributor or even a causative agent in human diseases which are called “conformational diseases” [3]. These include a diverse array of pathologies such as lysosomal storage diseases [4], cystic fibrosis [5] and many neurodegenerative disorders [6, 7]. To minimize the detrimental effects that misfolded and aggregated proteins impose, cells have evolved efficient protein quality control (PQC) systems to maintain proteostasis, which consist of the ubiquitin-proteasome system (UPS), chaperone mediated autophagy (CMA) and autophagy [8].

UPS is the major selective proteolytic system in eukaryotic cells, which degrades short-lived regulatory proteins and soluble misfolded proteins [9]. The conjugation of a polyubiquitin chain to target proteins is an essential step for their degradation by the 26S proteasome. Increasing evidences show that impaired and/or decreased function of the UPS is associated with many neurodegenerative diseases including Alzheimer’s disease (AD) [10], Parkinson’s disease (PD) and Huntington’s disease (HD) [11]. In addition to disease-causing proteins (e.g., amyloid β, alpha-synuclein, or Huntingtin), there are often disruptions in the Ubiquitin B gene (UBB) and mRNA transcripts, as well as polyubiquitin depositions within aggregates made of disease-specific proteins. UBB^+1^ is generated from a dinucleotide loss in the transcript due to RNA polymerase “slippage” during the transcription of the UBB gene, a process termed “molecular misreading”. The hotspots for molecular misreading are near short repeat sequence, such as the GAGAG motif [12]. The result of misreading is a frameshift near 3’ end of UBB mRNA transcript resulting in UBB^+1^, a UBB peptide variant with additional 20 amino acids at the C-terminus. Unlike the UBB, UBB^+1^ fails to ligate protein substrates or join polyubiquitin chains due to the absence of the C-terminal glycine residue, but like any other damaged protein recognized by the UPS system, it is readily ubiquitylated and degraded [13, 14].

AD is the most common form of neurodegeneration in aging population [15]. The accumulation of amyloid-β (Aβ) plaques in the brain is one of principal hallmarks of AD, which is thought to trigger a cascade of pathogenic processes [16]. Accumulation of UBB^+1^ is a cellular hallmark of sporadic and autosomal AD cases, suggesting its pathological contribution [17, 18]. The presence of UBB^+1^ has been proposed as an endogenous reporter for decreased UPS activity [19]. Previous studies showed that UBB^+1^ acts as a ubiquitin-fusion-degradation substrate for the proteasome and its properties shift from substrate to inhibitor, in a dose-dependent manner [14, 20]. Low levels of UBB^+1^ can be ubiquitinated and efficiently degraded by the UPS, whereas at high levels, the UPS fails to degrade UBB^+1^ and the accumulation of UBB^+1^ further induces functional impairment of the UPS. Prolonged expression of high levels of UBB^+1^ affects mitochondrial dynamics and triggers neuronal cell death [21, 22]. Despite the UBB^+1^-induced UPS dysfunction, in some cases UBB^+1^ expression is protective by the induction of heat-shock proteins, which promote cellular resistance to oxidative stress [23, 24]. UBB^+1^ expression reduces the Aβ plaque load in APPPS1 mice during aging through restoration of PS1-NTF expression and γ-secretase activity [25].

Although the impact of UBB^+1^ has been studied in different *in vivo* model systems, the precise role of UBB^+1^ in UPS dysfunction and its importance during AD progression remains ambiguous. The yeast *Saccharomyces cerevisiae* is a powerful eukaryotic model often used to study misfolded proteins and their implication in human pathologies due to the strong conservation of PQC systems between yeast and human cells [26]. To exploit the effects of UBB^+1^ expression on proteasome function and cellular viability, we recently developed two yeast models using constitutive expression of the human UBB^+1^, expressed at high and low levels [27]. We found that at low expression level, UBB^+1^ enhances cellular resistance to misfolded proteins and oxidative stress during chronological aging, and prolongs chronological life span (CLS), which measures the survival time of nondividing cells [27]. Aβ42 and Aβ40 are two major isoforms of Aβ associated with AD. Aβ40 is found in higher quantities in the affected brain tissue, but Aβ42 is more hydrophobic and more prone to aggregation. To mimic the chronic cytotoxicity of Aβ isoforms accumulation in AD progression, we have developed two humanized yeast AD models with Aβ42 and Aβ40 expression, respectively [28, 29]. These models have been used as a platform for synthetic genetic array (SGA) to screen for modulators of Aβ42 toxicity [30].

Here we take advantage of our low UBB^+1^ expression strain (hereafter referred to as L-UBB^+1^ strain) to investigate the underlying mechanisms that protect cells from stresses that we have previously identified [27] by using the genome-wide transcriptional analyses, followed by several molecular and cell biology assays. Transcriptome analyses helped to generate the hypotheses which were tested, which then led us to that low UBB^+1^ expression activated the autophagy pathway, which then reduced intracellular Aβ levels and alleviated its cellular toxicity.

## RESULTS

### Low expression of human UBB^+1^ significantly modifies the transcription of thousands of genes

**Figure 1:**
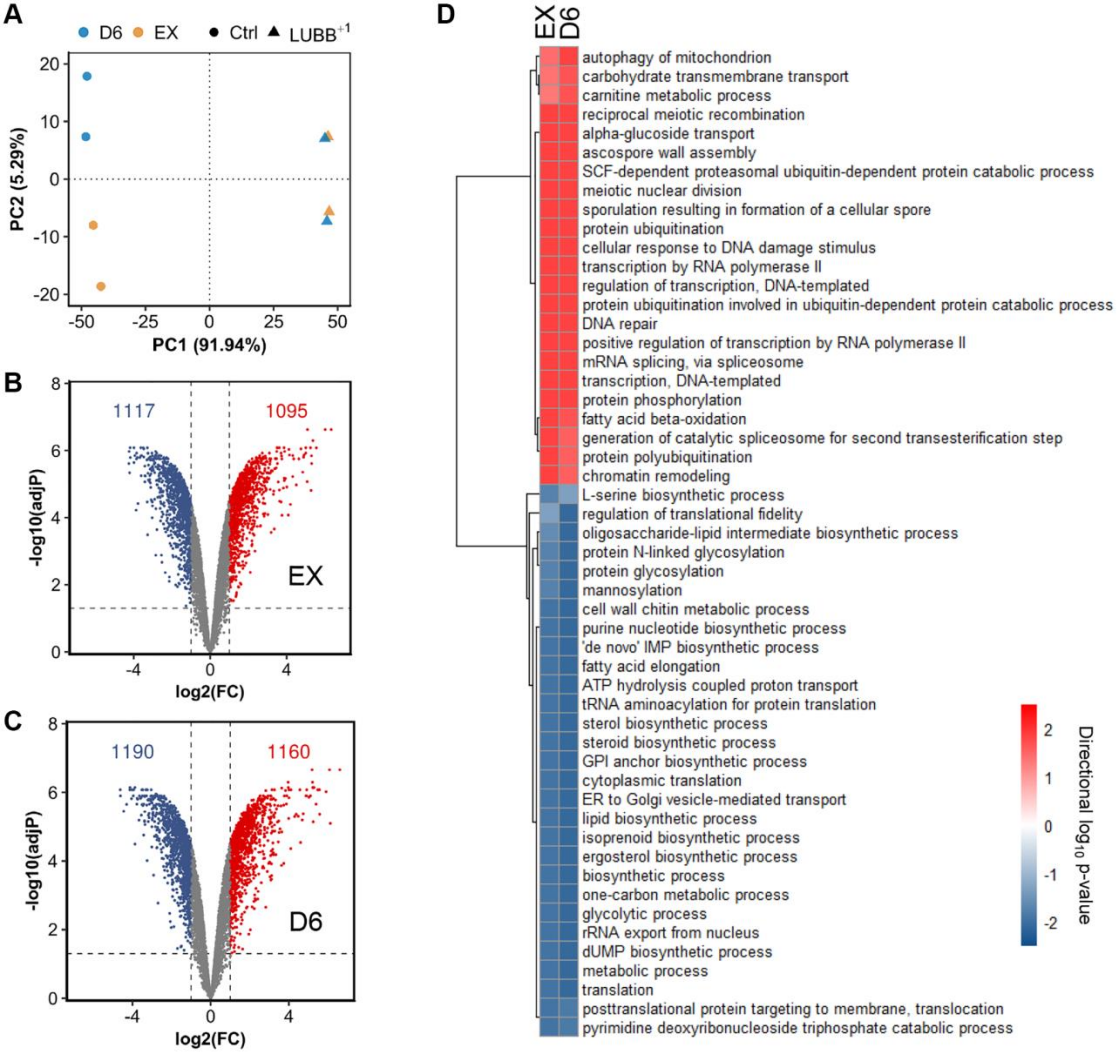
**The global transcriptional response to constitutively low UBB^+1^ expression.** (**A**) Principal Component Analysis (PCA) of the normalized microarray data. (**B**–**C**) Volcano plot of log_2_(FC) (Fold change) vs adjusted *p* value of differentially expressed genes comparing L-UBB^+1^ strain and control strain during EX (B) and D6 (**C**). The dashed vertical grey line indicates the threshold of log_2_(FC) (≤ -1 or ≥ 1), while the horizontal grey line indicates statistical significance threshold of adjusted *p* value < 0.05. (**D**) The significantly enriched GO terms in L-UBB^+1^ strain compared to control strain during EX and D6 phases. The red color indicates upregulated processes and blue color indicates downregulated processes. Samples are biological duplicates.

We have previously shown that at low expression levels, UBB^+1^ can extend CLS and increase cellular tolerance to misfolded proteins in yeast [27]. To investigate the mechanisms behind the observed phenotypes, we further performed a genome-wide transcriptional study and compared the gene expression between the control strain (carrying an empty vector) and the L-UBB^+1^ strain, during the exponential growth phase (EX) and stationary phase (D6, i.e., 6 days after carbon source in the medium has been used up). The principal component analysis (PCA) showed distinct gene expression profile between control strain and L-UBB^+1^ strain (Figure 1A and Supplementary Figure 1). Pair-wise comparisons of L-UBB^+1^ strain and control strain revealed that 2212 and 2350 genes were significantly differentially expressed (adj-*P* < 0.001 and log_2_FC ≤ -1 or log_2_FC ≥ 1) during EX and D6, respectively (Figure 1B and 1C). 1913 genes (72.2%) were significantly changed during both EX and D6 phases.

To gain more insight into biological processes affected by L-UBB^+1^ expression, we also performed the gene set enrichment analysis (GSA) on the significantly differentially expressed genes. In the L-UBB^+1^ strain, 23 and 29 gene sets were significantly upregulated and downregulated, respectively, in EX and D6 phases, compared to the control strain (adj-*P* < 0.05, Figure 1D). Gene sets associated with autophagy and ubiquitin-related processes, such as “protein ubiquitination”, “ubiquitin-dependent protein catabolism”, “SCF-dependent proteasomal protein catabolism” and “ubiquitin-protein transferase activity”, were enriched among upregulated genes in the L-UBB^+1^ strain. Our previous study showed the L-UBB^+1^ expression inhibits proteolytic activities of 20S proteasome [27]. The inhibition of proteasome results in the compensatory activation of UPS and autophagy [31], which is in accordance with our genome-wide transcriptional analysis results. Gene sets related to transcription, such as “DNA-templated transcription”, “transcription by RNA polymerase II”, “positive regulation of transcription by RNA polymerase II”, were enriched among upregulated genes as well (adj-*P* < 0.05, Figure 1D and Supplementary Figures 2 and 3). Whereas gene sets related to protein synthesis pathways, such as “translation”, “protein glycosylation”, “GPI anchor biosynthetic process”, “ER to Golgi transport”, “translocation”, were enriched among downregulated genes in the L-UBB^+1^ strain (adj-*P* < 0.05, Figure 1D and Supplementary Figures 2 and 3), which may alleviate the ER stress by reducing the influx of newly synthesized proteins into ER. In addition to these protein syntheses and processing related processes, genes related to metabolic process, such as “lipid biosynthetic process”, “nucleotide biosynthetic processes” and “glycolytic process”, were significantly downregulated in the L-UBB^+1^ strain (adj-*P* < 0.05, Figure 1D and Supplementary Figures 2 and 3).

### Low expression of human UBB^+1^ significantly increases the transcription of autophagy genes

**Figure 2:**
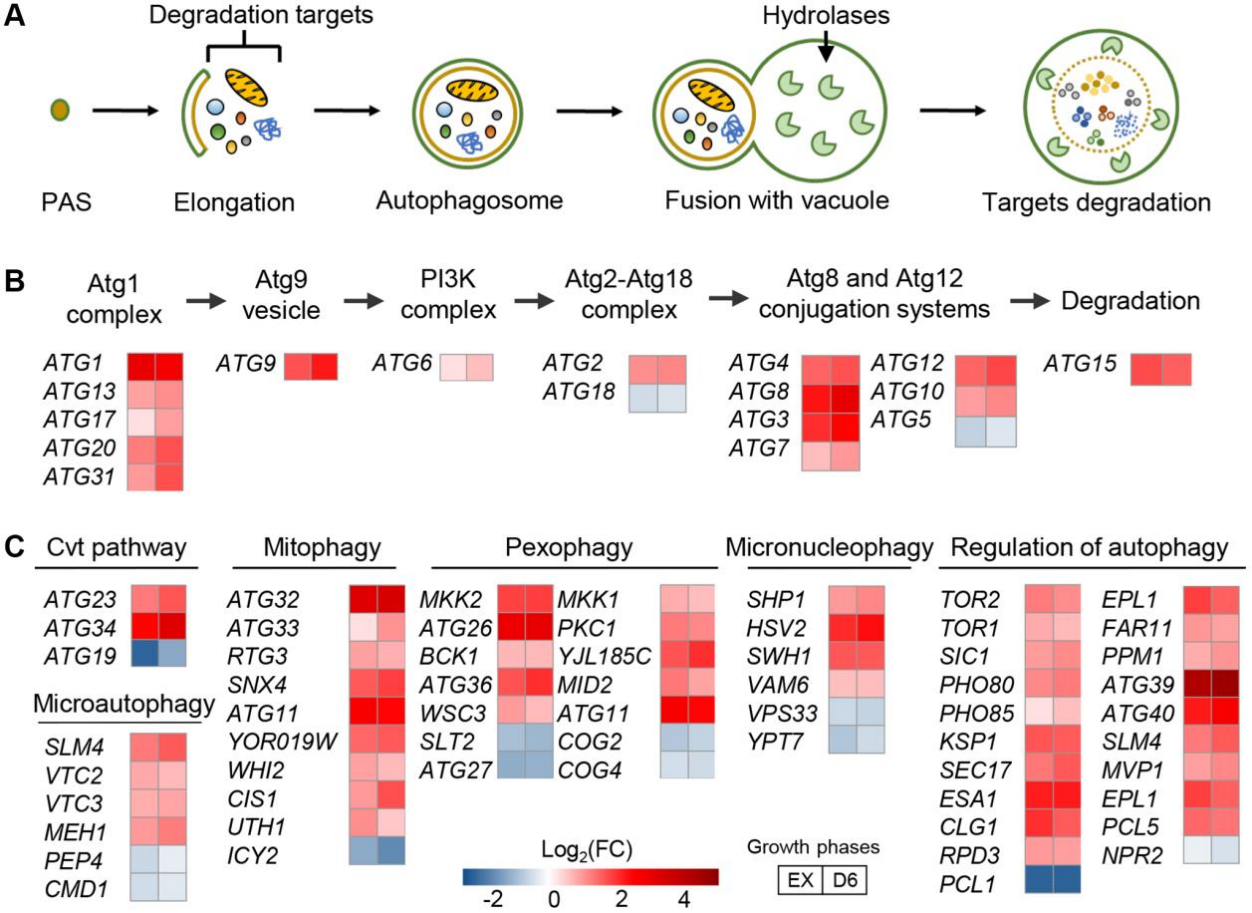
**Low UBB^+1^ expression activates autophagy at the transcript level.** (**A**) Schematic overview of autophagosome formation. (**B**) Fold changes in the expression of ATG genes encoding for autophagosome formation. Abbreviation: PI3K, phosphatidylinositol 3-kinase. (**C**) Fold changes in the expression of genes encoding different modes of autophagy. All comparison is between L-UBB^+1^ strain and control strain during EX and D6 phases (adj-*p* < 0.05).

Gene sets related to autophagy processes were significantly upregulated in the L-UBB^+1^ strain (Figure 2 and Supplementary Table 1). Autophagy is a major catabolic pathway which critically secures eukaryotic cellular homeostasis and survival [32]. Activation of autophagy extends the lifespan of many other model systems such as the nematode *Caenorhabditis elegans* [33], fruit fly *Drosophila melanogaster* [34] and mice [35], and protects cells against a variety of stresses [36, 37]. Macroautophagy is the most prevalent form of autophagy in which double-membrane structures called the autophagosomes are formed around cargoes designated for degradation, such as aberrant organelles and misfolded/aggregated proteins [38]. It starts with the appearance of an isolated membrane termed the pre-autophagosomal structure [39], which expands and seals itself into an autophagosome while engulfing bulk portions of cytoplasm. Upon fusion with the vacuole, the inner autophagosome contents are degraded by lysosomal hydrolases (Figure 2A). About 35 autophagy-related genes (ATG) have been identified in yeast [40]. Among these, 18 ATG genes in six functional groups are required for autophagosome formation: the Atg1 complex, Atg9, the autophagy-specific phosphatidylinositol 3-kinase (PI3K) complex, the Atg2-Atg18 complex, and the Atg8 and Atg12 conjugation systems [41]. Compared to the control strain, 15 out of these 18 ATG genes were found significantly upregulated in the L-UBB^+1^ strain (adj-*P* < 0.05, Figure 2B). The expression level of *ATG1*, an essential regulator required for the formation of the autophagosome in yeast [42], was 7.03 and 5.86-fold higher in the L-UBB^+1^ strain during the EX phase and D6 phase, respectively (Supplementary Table 2). qPCR (quantitative PCR) analysis verified that the transcript level of *ATG1* was 7.33-fold higher in L-UBB^+1^ strain during EX phase (*p* < 0.001, Supplementary Figure 4).

Most genes involved in “regulation of autophagy” and “cvt pathway”, “pexophagy”, “mitophagy” and “micronucleophagy” were also significantly upregulated in the L-UBB^+1^ strain (Figure 2C). Higher transcription levels of 10 autophagy related genes were further verified by qPCR analysis (Supplementary Figure 5). For illustration, 81 differentially expressed genes involved in autophagy related processes are listed in Supplementary Table 2.

### Low expression of human UBB^+1^ activates autophagy

**Figure 3:**
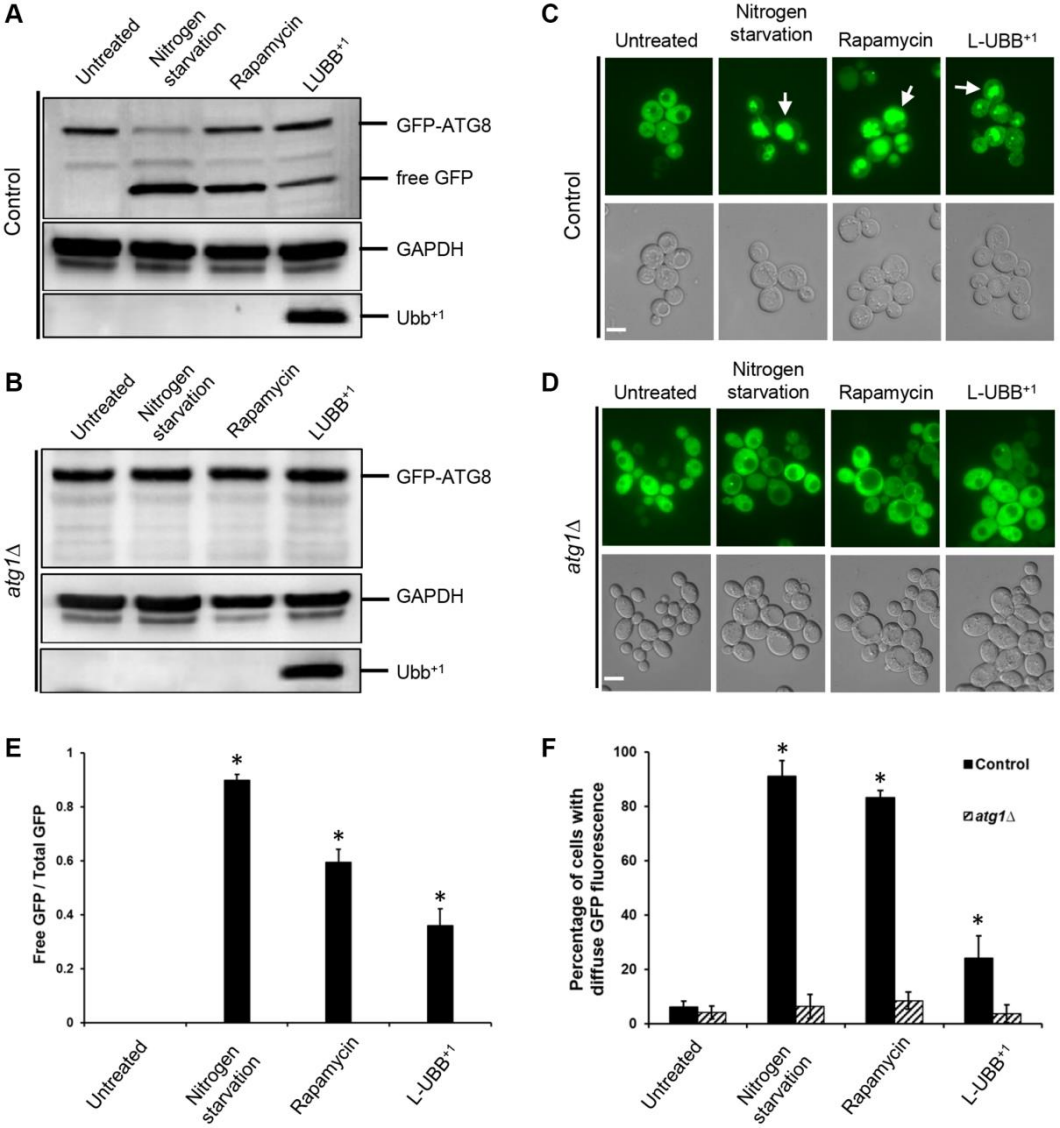
**Low UBB^+1^ expression activates autophagy.** (**A**–**B**) Western blot of GFP-Atg8p processing into free GFP. GAPDH was used as the loading control. (**C**–**D**) Translocation of GFP-Atg8p into yeast vacuole. Top panel: images from FLUO-GFP filter. Bottom panel: images from DIC filter. White arrow: GFP fluorescence inside vacuole. Scale bar = 5 μm. (**E**) The ratio of free GFP to total GFP (uncleaved GFP-ATG8 + free GFP) under wild type background was calculated and presented based on (**A**). Data is shown as average values ± SD from biological triplicates. (**F**) The percentage of cells with diffuse vacuolar GFP fluorescence was counted and represented based on (**C**–**D**). Above 200 cells were count per sample (*n* = 3 ± SD). The asterisk (^*^) indicates a statistically significant *p*-value of < 0.05 from untreated control strain.

To investigate whether the expression of L-UBB^+1^ led to an actual activation of the autophagy pathway, autophagy was monitored by measuring the cleavage of a GFP-Atg8 fusion protein. Atg8p is a protein essential for autophagy, which is transported to the vacuole for degradation during autophagy. The proteolysis of GFP-Atg8 releases an intact GFP, which can be detected and correlated with the autophagic rate [43]. The cleavage of GFP-Atg8 was assessed at mid EX phase in both control strain and L-UBB^+1^ strain. No cleavage of GFP was observed in control strain. In contrast, 36% of free GFP was detected in the L-UBB^+1^ strain, indicating the activation of autophagy (Figure 3A and 3E). Nitrogen starvation and rapamycin treatment are two known activators of autophagy [44], which resulted in 90% and 59% of free GFP cleavage in our control strain, respectively (Figure 3A and 3E). When we analyzed the GFP-Atg8 cleavage in autophagy deficient mutant (*atg1*Δ) background, no GFP-Atg8 cleavage was observed in the *atg1*Δ_L-UBB^+1^ strain, similar to the results from nitrogen starvation and rapamycin treatment in *atg1*Δ_control strain (Figure 3B). This indicates that the Atg1p is involved in L-UBB^+1^-induced activation of autophagy. Fluorescent microscopy was used to study the localization of GFP-Atg8p. Since GFP is relatively resistant to degradation, it accumulates in the vacuole as autophagy proceeds. In the L-UBB^+1^ strain, 24.1% of cells showed diffused GFP fluorescence in the vacuole (Figure 3C and 3F), which was significantly higher than 6% in the control strain (Figure 3C and 3F). The nitrogen starvation and rapamycin treatments in control strain showed respectively 91.1% and 83.2% of cells with stronger GFP fluorescence inside the vacuoles (Figure 3C and 3F). In the *atg1*Δ mutant strain, the accumulation of GFP fluorescence in the vacuole was absent under the same treatments (Figure 3D and 3F), revealing the inability of mutant cells to activate autophagy.

**Figure 4:**
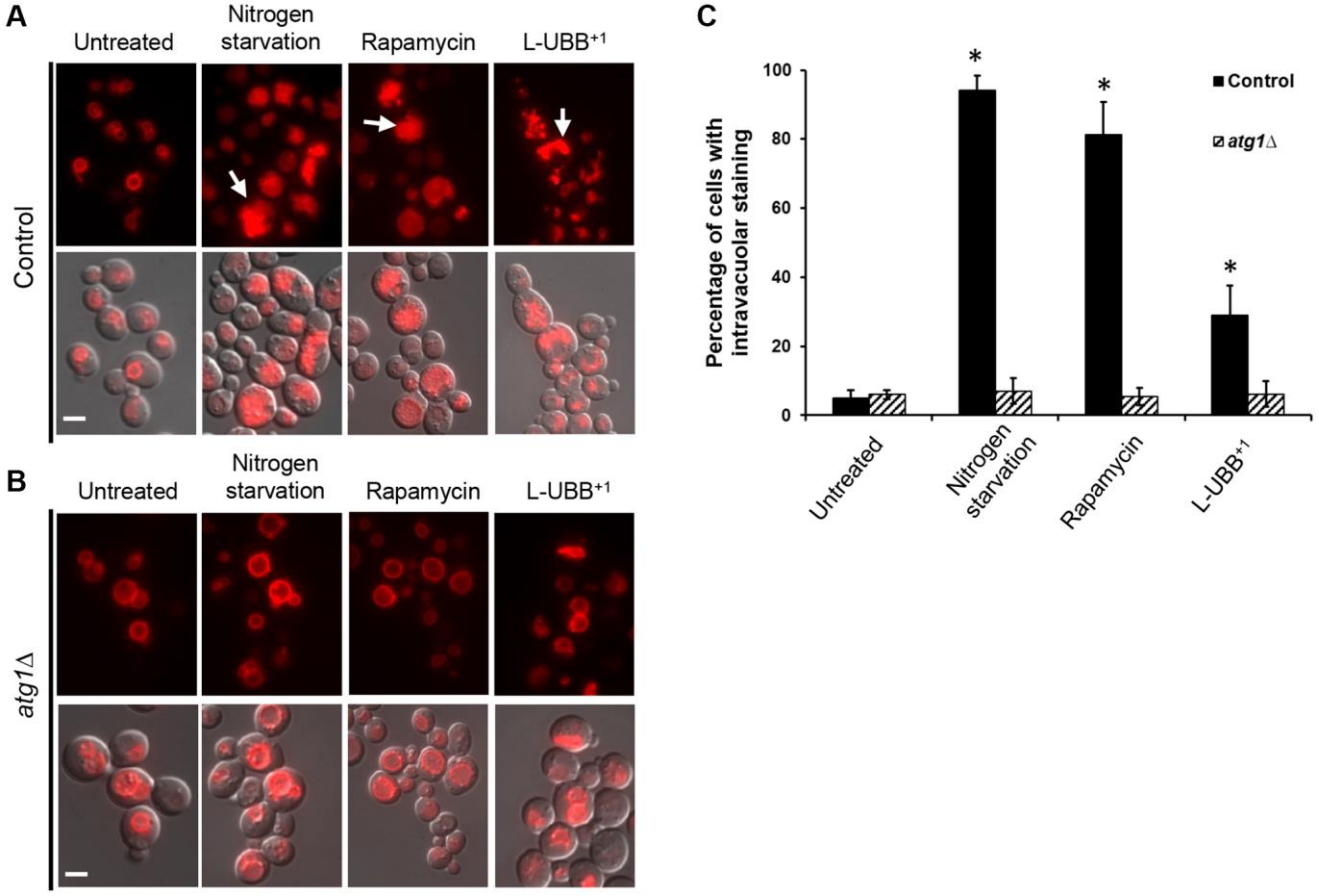
**Low UBB^+1^ expression increases vacuolar activity.** Images of vacuole staining with FM 4-64 under wild type background (**A**) and *atg1Δ* mutant background (**B**). Top panel: FM 4-64 fluorescence. Bottom panel: overlay of DIC and FM 4-64 fluorescence images. White arrows indicate cells with intravacuolar staining. Scale bar = 5 μm. (**C**) Quantification of the percentage of cells containing intravacuolar staining in the indicated strains. The data are shown as average values ± SD from three independent experiments, with more than 200 cells per experiment. The asterisk (^*^) indicates significant differences from the untreated control strain (*p* < 0.05).

Autophagy was also monitored by following the bulk transport of cytosolic contents to vacuole for degradation using a FM 4-64 dye [45]. In the absence of autophagy, only the vacuolar perimeter was stained with FM 4-64 (Figure 4A). Under autophagy-induced conditions, cells showed intravacuolar staining and multivesicular bodies. Nitrogen starvation and rapamycin treatment resulted in 94.1% and 81.2% of cells showing such intravacuolar staining, respectively (Figure 4A and 4C). For the L-UBB^+1^ strain, 29% of the cell population showed intravacuolar staining (Figure 4A and 4C), which was significantly higher than the control strain (*p* < 0.05). In the *atg1*Δ mutant background, there was no significant intravacuolar staining neither with L-UBB^+1^ expression, nor under nitrogen starvation and rapamycin treatment (Figure 4B and 4C).

### Low expression of human UBB^+1^ significantly extends chronological life span

**Figure 5:**
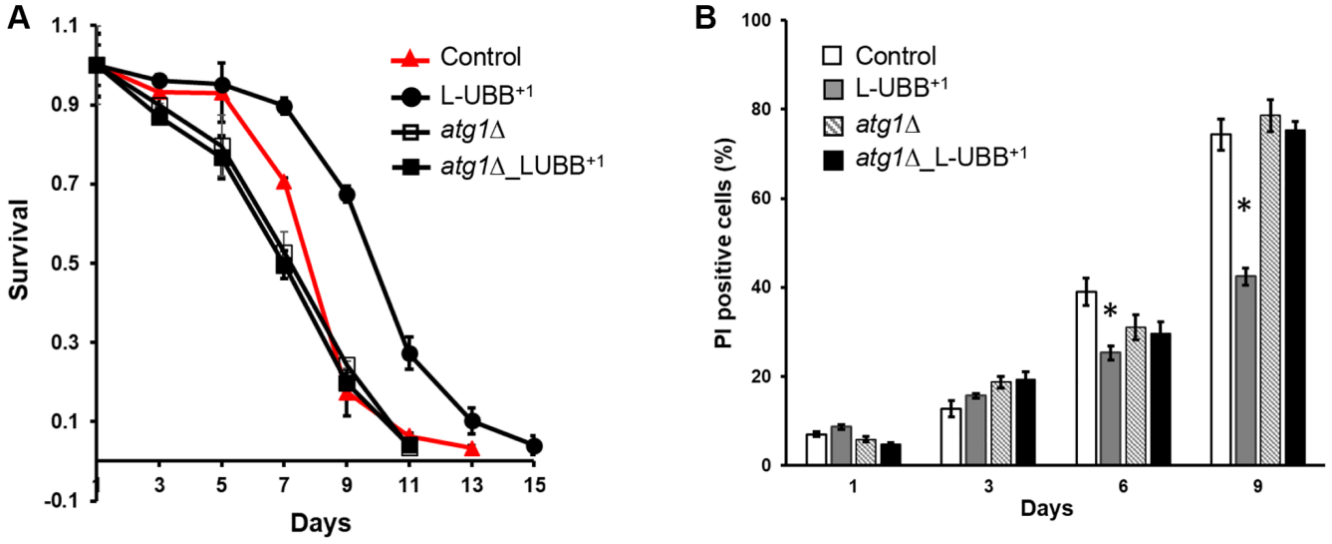
**Low UBB^+1^ expression extends *ATG1*-dependent CLS.** (**A**) Survival of the L-UBB^+1^ strain during stationary phase under wild type background and *atg1Δ* mutant background. Viability was determined by CFU counting. (**B**) Percentages of dead cells are shown as the fraction of propidium iodine (PI) positive cells. The data are shown as mean ± SD from biological duplicates. ^*^p < 0.01.

Beyond its function in turn-over and renewal of cellular contents, autophagy plays a prominent role in the life span of many model organisms. Multiple reports indicate that a plethora of nutritional, pharmacological, or genetic manipulations that increase life span often stimulate autophagy, whereas inhibition of autophagy is associated with accelerated aging [34, 46, 47]. To determine whether the L-UBB^+1^ expression-induced autophagy led to alterations in life span, we performed CLS analyses to the control, L-UBB^+1^, *atg1*Δ and *atg1*Δ_L-UBB^+1^ strains. The number of surviving cells were determined by colony forming unit (CFU) counting (Figure 5A) and PI staining (Figure 5B). Compared to the control strain, the L-UBB^+1^ strain displayed a significantly greater survival after 5 days and the CLS extended from 13 days to 15 days (*p* < 0.01, Figure 5A). However, this markedly extended life span was abrogated when *ATG1* was deleted and life span was shortened to 11 days in *atg1*Δ_L-UBB^+1^ strain (Figure 5A). The source data for Figure 5A was provided in Supplementary Table 3. In accordance with this, the PI staining showed significantly decreased fractions of dead cells in L-UBB^+1^ culture on day 6 and day 9 compared to the control strain (*p* < 0.01, Figure 5B). The fraction of dead cells was 32% lower in L-UBB^+1^ strain than control strain at day 9 (Figure 5B).

### Low expression of human UBB^+1^ significantly reduces Aβ levels and cytotoxicity

**Figure 6:**
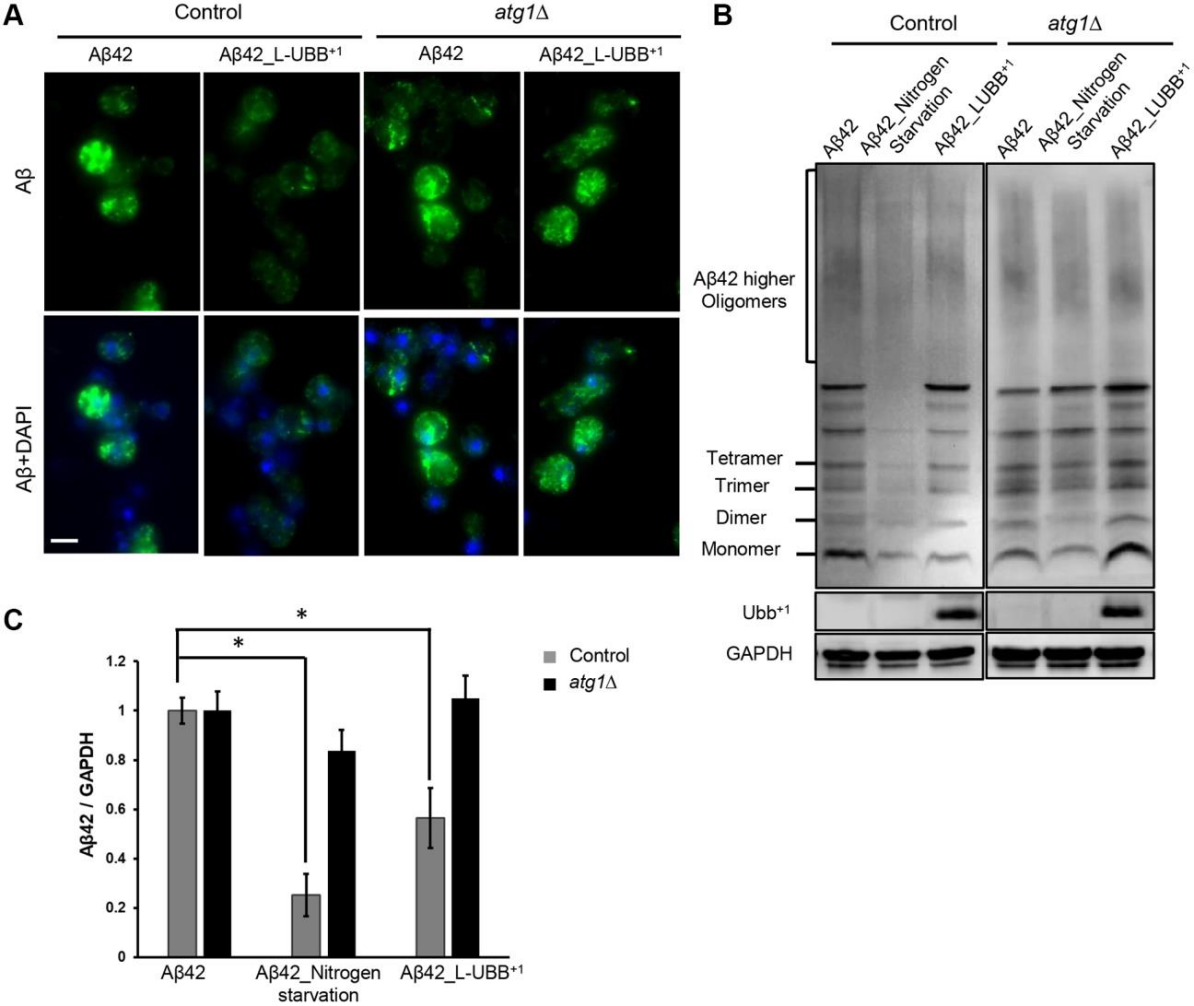
**Low UBB^+1^ expression reduces Aβ42 levels in the humanized yeast AD model.** (**A**) Immunostaining analysis of Aβ42 localization and expression using the 6E10 Aβ specific antibody. Nuclei were stained blue by DAPI. Scale bar = 5 μm. (**B**) Western blot analysis of Aβ42 expression in unboiled cell lysates with 6E10 antibody. GAPDH was used as the loading control. (**C**) Relative Aβ42 band intensity was normalized to GAPDH and compared to the untreated Aβ42 strain. Results are reported as mean ± SD of three independent experiments. ^*^p < 0.05.

In previous study, we developed yeast Aβ models that mimic the chronic cytotoxicity of the amyloid peptides [28]. The expression of two major Aβ peptides, Aβ40 and Aβ42, interferes with cellular metabolism and causes different levels of ER stress which regulate cell fate [29]. Here we took advantage of these established Aβ models to investigate whether the L-UBB^+1^ expression could affect the different Aβ toxic isoforms. Immunostaining confirmed the localization of Aβ in the ER/secretory compartment (Figure 6A and Supplementary Figure 6A). In the Aβ42 expression strain, Aβ concentrated in small foci (Figure 6A), compared to a more disperse distribution in the Aβ40 strain (Supplementary Figure 6A), as we discovered previously [28]. Aβ oligomers were detected in the Aβ42 strain (Figure 6B) when protein lysates were not subjected to boiling, which disrupts the oligomers. In the Aβ40 strain, only monomer and dimer were observed in unboiled samples (Supplementary Figure 6B). This clearly illustrates the different capacity of both peptides to form aggregates. When L-UBB^+1^ was co-expressed in the Aβ42 and Aβ40 strains, a significant reduction in the immunostaining fluorescence was observed in both Aβ42 (Figure 6A) and Aβ40 strains (Supplementary Figure 6A). L-UBB^+1^ expression significantly decreased Aβ levels in the Aβ42 strain (*p* < 0.05, Figure 6B and 6C) as determined by immunoblotting. The Aβ40 strain was less sensitive to L-UBB^+1^ expression, which led to a milder reduction of Aβ40 levels (*p* < 0.05, Supplementary Figure 6B and 6C).

The reduced intracellular Aβ42 and Aβ40 levels might in part be due to enhanced autophagy upon L-UBB^+1^ expression. In the *atg1*Δ mutant strain, L-UBB^+1^ co-expression did not significantly alter Aβ levels in neither Aβ42 strain (Figure 6) nor Aβ40 strain (Supplementary Figure 6), indicating that activated autophagy was important for reduced Aβ levels upon L-UBB^+1^ expression.

**Figure 7:**
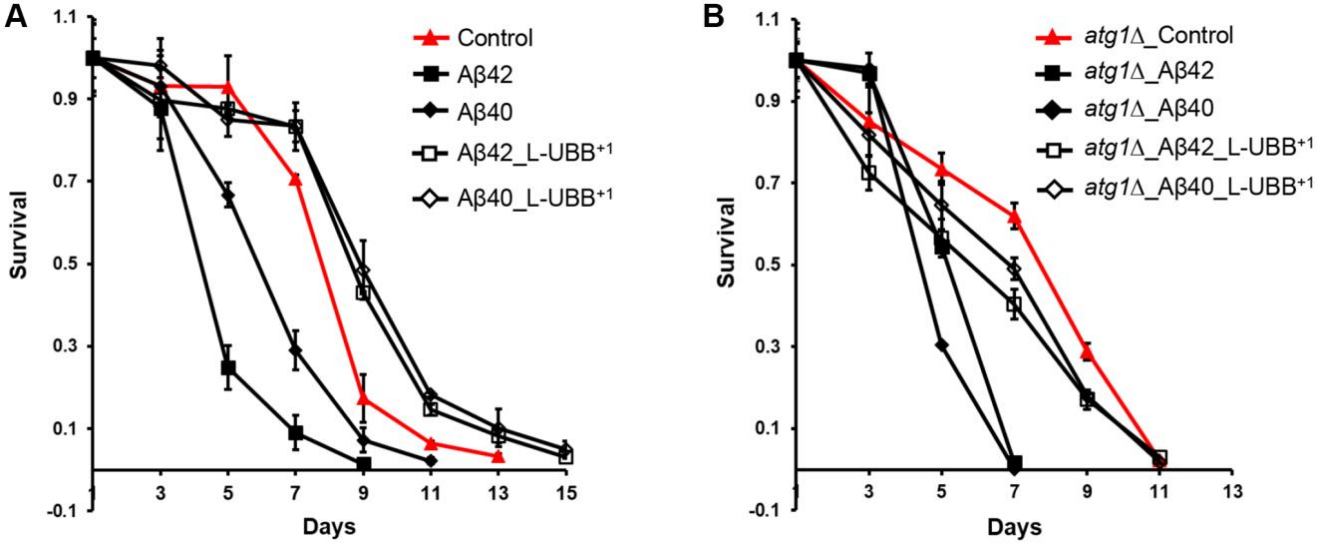
**Low UBB^+1^ expression reduces Aβ42 and Aβ40 toxicity.** (**A**) Survival of the Aβ42 and Aβ40 strains during stationary phase without or with low UBB^+1^ expression under wild type background. (**B**) Survival of the Aβ42 and Aβ40 strains during stationary phase without or with low UBB^+1^ expression under *atg1Δ* mutant background. Viability was determined by CFU counting. The data are shown as mean ± SD from biological duplicates.

The Aβ42 strain displayed a 17% reduction of maximal specific growth rate, compared to the control strain, and a shortened CLS of 9 days compared to 13 days in the control strain (Figure 7A), in agreement with our previous observations [28]. L-UBB^+1^ expression did not restore the decreased maximal specific growth rate of the Aβ42 strain (data not shown), however it did significantly enhance the cell survival. The CLS was extended to 15 days in the Aβ42_L-UBB^+1^ strain compared to 9 days in the Aβ42 strain (Figure 7A). Although the Aβ40 strain did not show the notable differences in physiology from control strain [29], CLS was shortened to 11 days compared to 13 days in the control strain. L-UBB^+1^ co-expression also led to an extended CLS in the Aβ40 strain, which showed a similar viability pattern with the Aβ42_L-UBB^+1^ strain (Figure 7A).

The *atg1*Δ mutant strain showed a shorter CLS compared to the control strain (Figure 7B). Deficient expression of *ATG1* (Unc-51) has been shown to decrease the life span of *C. elegans* [48] and *D.melanogaster* [49]. Aβ42 and Aβ40 expression in an *atg1*Δ mutant background led to a similar and remarkably shorter CLS of 7 days (Figure 7B), indicating that absence of autophagy increases cellular susceptibility to Aβ toxicity. L-UBB^+1^ co-expression with Aβ42 or Aβ40 in the *atg1*Δ background strain increased cell survival (Figure 7B), however the effect was not as strong as that of co-expression in the wild type background (Figure 7A). The CLS was extended to 11 days in both *atg1Δ*_Aβ42_L-UBB^+1^ strain and *atg1Δ*_ Aβ40_L-UBB^+1^ strain, with lower survival compared to *atg1*Δ mutant strain. This suggests that the Aβ toxicity attenuation by low UBB^+1^ expression is not solely determined by elevated autophagy, but probably involves a secondary mechanism. The source data for Figure 7 was provided in Supplementary Table 3.

## DISCUSSION

Humanized yeast models have been constructed and used to investigate molecular mechanisms underlying several human neurodegenerative disorders, by expressing human proteins implicated (or suspected to play a relevant role) in these diseases and studying the effects on yeast cell physiology, fitness, and different molecular pathways [50, 51]. UBB^+1^ has been found to accumulate in the brain of AD patients [52] and it is thus believed that it might contribute to the development of neuropathology of AD [53], thus a humanized yeast model by using heterologous expression of UBB^+1^ in yeast, could provide insights into its role(s) *in vivo*. We found that constitutive low levels of UBB^+1^ expression increase the capacity to degrade misfolded proteins and prevent cells to accumulate reactive oxygen species [27]. Here, we investigated the potential molecular mechanisms behind these effects by using genome-wide transcriptional analyses to generate hypotheses, which we tested by using molecular and cell biology tests. We found that the autophagy pathway was significantly upregulated in L-UBB^+1^ strain, which may therefore contribute to decreased intracellular Aβ42 and Aβ40 levels and attenuated Aβ-induced cytotoxicity.

Autophagy is an evolutionarily conserved catabolic pathway used to degrade misfolded or aggregated proteins, as well as damaged cellular organelles, and is an important neuroprotective mechanism [54–56]. Neurons and glia in the central nervous system (CNS) are highly specialized post-mitotic cells that need to continuously remove defective proteins and organelles [57]. Cellular and animal models have shown that autophagy pathways are involved in the regulation of neurogenesis, and if they are not functional lead to neuronal disorders. Deficient autophagy in microglia results in impaired synaptic refinement and social behavioral defects [58, 59].

Our transcriptional analyses revealed that low expression of UBB^+1^ elevated the expression of genes involved in ubiquitin-related processes and autophagy pathways. Genes involved in macroautophagy and selective autophagy pathways were significantly upregulated (Figures 1 and 2). In addition, UBB^+1^ expression increased the intravacuolar accumulation of FM4-64 stained vesicles after PMSF treatment, indicating increased vacuolar activity (Figure 4). Analysis of distribution and cleavage of GFP-Atg8 showed that UBB^+1^ expression promoted the uptake of Atg8 into vacuole and the cleavage of free GFP from the GFP-Atg8 fusion (Figure 3). This process occurs during autophagy where GFP-Atg8 is engulfed by the completed autophagosomes and then degraded [60]. Similar effects were observed with two known strong activators of autophagy, nitrogen starvation and rapamycin treatment (Figures 3 and 4), suggesting that low UBB^+1^ expression increases autophagy activity, but moderately. Impaired autophagy with reduced capacity to eliminate pathogenic proteins has been reported in many neurodegenerative disorders such as AD and PD [61]. Autophagy lysosomes are increased in early stages of AD, whereas impaired clearance of autophagic vesicles, e.g., maturation and transport of autophagosomes, and reduced lysosomal proteolysis, are observed in later stages of AD, which may contribute to Aβ accumulation [62, 63]. Activating autophagy by rapamycin treatment, an inhibitor of mTOR pathway, protects neuroblastoma cells from Aβ toxicity [64], reduces cerebral Aβ load and slows AD progression in a transgenic AD mouse model [65]. Our data showed that low UBB^+1^ expression reduced intracellular levels of Aβ42 and Aβ40 in the wild type background but not in the *atg1*Δ mutant background (Figure 6 and Supplementary Figure 6), indicating that L-UBB^+1^ expression decreased Aβ levels as a function of autophagy activation. Activation of autophagy has been shown to protect cells against multiple forms of stress, including nutrient and growth factor deprivation, reactive oxygen species, endoplasmic reticulum stress, damaged organelles or protein aggregates [66]. We observed that the low UBB^+1^ expression prolonged CLS in Aβ strains during chronological aging. The increased cell survival was reverted in the *atg1*Δ mutant background, further supporting the notion that activation of autophagy is crucial in promoting cellular survival and protection against Aβ induced toxicity.

Besides the autophagy pathways, the genome-wide transcriptional analyses also revealed that many UPS-related processes were activated in response to low UBB^+1^ expression. The UPS is a key component of the PQC for maintaining the proper concentrations of many regulatory proteins and clearing damaged/misfolded proteins [67]. Several studies suggest that sustained proteasome activity correlated with longevity, as found in centenarians [68], immortal cells such as human Embryonic Stem Cells (hESCs) [69], long-lived animals such as the naked mole-rat [70] and the giant clam [71]. The correlation has been further supported by genetic approaches. A genetic gain-of-function screening in *D. melanogaster* shows that *rpn11*, encoding a subunit of the 19S regulatory particle (RP), extends the flies’ life spans with suppression of accumulated ubiquitinated proteins during aging process [72]. Increased expression of *rpn6*, another subunit of the 19S RP, results in elevate proteasome activity, clearance of toxic PolyQ aggregated and increased longevity in *C. elegans* [73]. *Rpn4* is required to induce proteasome subunits under conditions of proteasome dysfunction [74] and elevated *rpn4* levels increase UPS capacity which enhances replicative lifespan and resistance to proteotoxic stress in yeast [75]. The expression levels of *rpn11*, *rpn6* and *rpn4* were significantly increased in the L-UBB^+1^ strain, which may additionally assist in reducing Aβ cytotoxicity.

UPS and autophagy are two major protein degradation systems in eukaryotic cells, which aim at maintaining proteostasis. Recent studies strongly suggest functional crosstalk and interplay between these two systems. Autophagy can be activated in response to genetic or pharmacological inhibition of UPS [76]. With impaired proteasome function, the aberrant protein aggregates form large inclusion body-like structures known as aggresomes [77], which are thought to promote autophagy-mediated degradation [39]. Compensatory autophagy was induced in response to a dysfunctional UPS in a *Drosophila* model of the spinobulbar muscular atrophy via a histone deacetylase 6 (HDAC6)-dependent aggresome pathway [78]. The molecular mechanisms underlying autophagy activation in response to UPS inhibition are not clear, but many factors may be involved, including the N-terminal arginylation of N-end rule pathway [79], the unfolded protein response [80], and the BCL family protein MCL1 (myeloid cell leukemia sequence 1) [81]. Previous studies have showed that UBB^+1^ is a dose-dependent inhibitor of UPS [20]. We found previously that the overexpression of UBB^+1^ indeed decreases the proteolytic activities of the proteasome [27].

Overall, our study shows that low UBB^+1^ expression significantly increased the autophagy activity and thus induced intracellular degradation of Aβ, which increased cell fitness and survival. Identifying how moderate induction of autophagy can significantly reduce Aβ accumulation and consequently reduce its cytotoxicity could be relevant for understanding better the molecular onset and progression of AD, as well as potential targets for pharmacological intervention.

## MATERIALS AND METHODS

### Strains and cultivation

The haploid laboratory strain *S. cerevisiae* CEN.PK113-11C (*MATα his3Δ1 ura3-52 MAL2-8c SUC2*) [82] was used as a reference strain in this study. The *atg1Δ* mutant strain was constructed by transforming the reference strain with a PCR amplified *KanMX* cassette (from the pUG6 plasmid [83]) including approximately 500 bp upstream sequence and 500 bp downstream sequence flanks homologous to the *ATG1* locus. The gene deletion was confirmed by PCR using primers outside the *ATG1* open reading frame (ORF) and inside the *KanMX* gene respectively. All primers used are listed in the Supplementary Table 4. The previously described p413 TEF-UBB^+1^, p416 GPD-Kar2-Aβ42 and p416 GPD-Kar2-Aβ40 plasmids for constitutive expression of UBB^+1^, Aβ42 and Aβ40 respectively [27, 28] were transformed into the reference strain and *atg1Δ* strain. The p413 TEF-EP plasmid [84] was transformed into the reference strain and *atg1Δ* strain to construct control strains. The pRS416 GFP-ATG8 expression plasmid containing the *GFP-Atg8* gene under the endogenous *ATG8* promoter was donated by Prof. Daniel Klionsky, University of Michigan [85] (http://www.addgene.org/49425/, RRID:Addgene 49425). All plasmids and yeast strains used in this study are summarized in Supplementary Table 5.

All yeast transformations were performed following a standard lithium acetate method and transformants were selected on synthetic dextrose (SD) medium without histidine for L-UBB^+1^ strain (SD-His, Formedium, England), or without uracil for Aβ42 and Aβ40 strains (SD-Ura, Formedium, England), or without both histidine and uracil for L-UBB^+1^ and Aβ42/Aβ40 co-expression strains (SD-His-Ura, Formedium, England). For cultivation, strains were grown in liquid SD medium with 20 g l^−1^ glucose. Synthetic minimal medium without ammonium sulfate and amino acids (YNB (-N) medium, Formedium) containing 20 g l^−1^ glucose was used for nitrogen starvation experiments.

### Transcriptome

Biological duplicate cultures from the control strain and L-UBB^+1^ strain were sampled during EX and D6 for microarray analysis. Cells were frozen immediately in liquid nitrogen for rapid quenching of mRNA turnover [86]. Cells were mechanically disrupted using a FastPrep homogenizer (MP Biomedicals, USA) and total RNA was extracted using the RNeasy Mini Kit (QIAGEN, Germany). Quality of total RNA was assessed using an RNA 6000 Nano LabChip Kit (Agilent Technologies, USA) with an Agilent 2100 Bioanalyzer (Agilent Technologies, USA). The labeled RNA was generated using the GeneChip^®^ 3′ IVT Plus Reagent Kit (Affymetrix, USA), which was hybridized to GeneChip^®^ Yeast Genome 2.0 Arrays (Affymetrix, USA). Staining and washing of the hybridized arrays were performed on the GeneChip^®^ Fluidics Station 450 (Affymetrix, USA). Further microarrays were scanned in GeneChip^®^ Scanner 7G (Affymetrix, USA). RNA labelling, array hybridization and scanning were performed by the Bioinformatics and Expression Analysis core facility at Karolinska Institute, Sweden. Microarray data are available at the Genome Expression Omnibus website (GEO, http://www.ncbi.nlm.nih.gov/geo/) with the accession numbers GSE129688. The transcriptome data (CEL files) were analyzed using the R version 3.4.0 and the PIANO package (Platform for Integrative Analysis of Omics) with information from the Saccharomyces Genome Database (https://www.yeastgenome.org/) [87]. Gene set enrichment analysis (GSA) was performed to identify overrepresentation of functional annotation categories using the Database for Annotation, Visualization and Integrated Discovery (David, https://david.ncifcrf.gov/). The S288C yeast genome background was used to analyze the magnitude of fold enrichment. The differential gene expression (log_2_-FC) and corresponding significance (adjusted *p*-value) were calculated by the Benjamini–Hochberg method. Heatmaps of significantly differentially expressed genes and gene sets were generated by pheatmap R package.

### Immunoblotting

Protein extraction and western blotting were performed as described previously [28]. 5 OD_600 nm_ of cells were spun down at 4000 g for 5 min. Cell pellets were resuspended in 200 μl of lysis buffer containing 50 mM HEPES (pH 7.5), 150 mM NaCI, 2.5 mM EDTA, 1% Triton X-100 with Complete Mini Protease Inhibitor (Roche, Switzerland). 200 μl of glass beads (MP Biomedicals, USA) was added to the solution, then the cells were mechanically disrupted for 3 min on the FastPrep homogenizer (MP Biomedicals, USA) at 4°C. Afterwards, samples were centrifuged at 13 000 g for 15 min at 4°C, and the supernatant was collected as lysate. Protein concentrations in the lysate were measured using a BCA protein assay kit (Thermo Scientific, USA) and 50 μg of protein for each sample was loaded on a 4–12% Bis-Tris gel (Invitrogen, USA). Primary antibodies 6E10 (anti-Aβ residues 1-16, Covance, USA), anti-GFP (Roche, Switzerland), anti-Ub^+1^ (Santa Cruz, USA) and anti-GAPDH (Santa Cruz, USA) were used for immunoblotting. Blots were developed using ECL Prime reagents (GE Healthcare, USA) and scanned by ChemiDoc MP Imaging System (BioRad, USA). Images were quantified with Image J.

### GFP-Atg8 processing assay

*S. cerevisiae* strains harboring the pRS416 GFP-Atg8 expression plasmid were grown to mid exponential phase (OD_600 nm_ 0.5–0.6) in SD-Ura-His medium. Cells were washed in PBS once and cultured in SD-Ura-His medium, YNB (-N) medium and SD-Ura-His medium with 0.2 μM rapamycin respectively for 4 h at 30°C. Following incubation, 5 OD_600 nm_ of cells were harvested for western blot analysis using anti-GFP antibody (Roche, Switzerland) and anti-GAPDH antibody (Santa Cruz, USA). The rest of cells were observed by Leica AF 6000 inverted fluorescence microscopy (Leica DMI4000B, Germany) using the DIC and FLUO-GFP filters. Images were processed with the Leica Application Suite (LAS) software.

### FM 4-64 staining

As a lipophilic styryl dye, FM 4-64 specifically stains the vacuolar membrane in yeast based on the method described by Journo D et al. in 2008 [45]. Yeast cells (control, L-UBB^+1^, *atg1Δ*_control and *atg1Δ*_L-UBB^+1^ strains) were cultured to mid exponential phase (OD_600 nm_ 0.5–0.6) in SD-His medium. 5 OD_600 nm_ units of cells were harvested and resuspended in 1 ml of YPD medium containing 4 μM of FM 4-64 dye (Invitrogen, USA). Cells were cultivated for 30 min at 30°C in the dark. Then cells were resuspended in 10 ml of YPD without FM 4**-**64 and incubated for 40 min at 30°C. After washing in 50 mM HEPES buffer (pH 7) twice, cells were resuspended in either SD-His medium or YNB (-N) medium containing 1 mM PMSF (Phenylmethylsulfonyl fluoride, Sigma Aldrich, USA) and 10 mM sodium citrate (pH 4.3). Rapamycin (MW 914.17, Cat no. R8781, Sigma Aldrich, USA) treatment was done in SD-His medium with a final concentration of 0.2 μM. After 4 h incubation at 30°C, cells were washed and resuspended in YNB (-N) medium containing 10 mM sodium citrate (pH 4.3) and visualized by Leica AF 6000 inverted fluorescence microscopy (Leica DMI4000B, Germany) using the DIC and FLUO-RFP filters. Images were processed with the Leica Application Suite (LAS) software and the numbers of cells with intravacuolar staining were quantified.

### Chronological Life Span (CLS) assay

CLS was determined as described previously [88]. Yeast strains were inoculated into 5 ml of SD-Ura, SD-His or SD-Ura-His medium depending on the strain requirements and grown overnight. After 20 h, cells were diluted into 20 ml of fresh SD medium to an initial OD_600 nm_ of 0.1. Cultures were grown under continuous shaking (200 rpm) at 30°C. After 48 h, maximal cell densities were reached and therefore this time point was considered as day 1. Subsequently, cellular viability was estimated by a CFU assay every two days until day 15. Approximately 400 cells were plated onto SD plates and incubated at 30°C for 48 h. CFU was calculated as the number of colonies formed divided by the number of plated cells.

### Propidium iodide staining

Cell death was measured by propidium iodide (PI, Thermo Fisher Scientific, USA) staining as previously described [28]. 0.5 OD_600 nm_ of cells were taken at different time points (e.g., 1-, 3-, 6- and 9-days) during cultivation. Cells were washed once at 4000 g for 5 min with PBS and stained with 0.5 μg ml^−1^ of PI for 20 min in the dark. 5000 cells were analyzed for each sample with Guava flow cytometer (Merck, Germany). Experiments were performed in biological triplicates.

### Immunostaining

Strains were grown in SD-Ura or SD-His medium overnight at 30°C. Cultures were diluted into 20 ml of SD medium (OD_600 nm_ 0.1) and grown to mid exponential phase (OD_600 nm_ 0.5–0.6). Cells were spun down and fixed immediately with 5 ml of 4% formaldehyde, 50 mM KPO_4_ (pH 6.5) and 1 mM MgCI_2_ for 2 h. After fixation, cells were washed in 5 ml of PM (0.1 M KPO_4_ pH 7.5 and 1 mM MgCI_2_) and resuspended in PMST (0.1 M KPO_4_ pH 7.5, 1 mM MgCI_2_, 1 M Sorbitol and 0.1% Triton X-100) to a final OD_600 nm_ of 10. 100 μl of cells were incubated with 0.6 μl of 2-mercaptoethanol and 1 mg ml^−1^ zymolyase (Zymo Research, USA) for 40 min at 37°C. Spheroplast suspension was added to a polylysine-coated cover glass. The cells were blocked in 0.5% BSA/PMST for 30 min at RT, and incubated with primary antibody (6E10, Covance, USA) overnight at 4°C. After rinsing 3 times with PMST, cells were incubated with secondary antibody (anti-mouse Alexa 488, Dako, Denmark) for 2 h at RT in the dark. Then cells were stained with 0.4 mg ml^−1^ DAPI (4′,6-diamidino-2-phenylindole) for 5 min in the dark. Images were acquired using Leica AF 6000 fluorescence microscope (Leica DMI4000B, Germany), and processed with LAS software.

### Real-time quantitative PCR (qPCR)

qPCR was performed as previously described [29]. 1 μg of total RNA was used for cDNA synthesis with the QuantiTect Reverse Transcription Kit (QIAGEN, Germany). 2 μl of synthesized cDNA was used as the template for qPCR reaction with a DyNAmo Flash SYBR Green qPCR kit (Thermo Fisher Scientific, USA). Threshold cycle (Ct) values were obtained and the ΔΔCt method was used to calculate the fold change in transcript levels. RNA levels were normalized to the housekeeping gene *ACT1*. The primer sets are listed in Supplementary Table 4.

### Statistical analysis

Significance of differences between strains were determined as mean ± SD using two-tailed student *t* tests. A *p-*value < 0.05 was considered statistically significant unless specified explicitly. All experiments were done in biological triplicates unless specified otherwise.

## Supplementary Materials

Supplementary Figures

Supplementary Tables
