# Supplementary material for: UBB+1 reduces amyloid-β cytotoxicity by activation of autophagy in yeast
Source: Aging (Albany NY). 2021 Nov 9;13(21):23953–80. doi: 10.18632/aging.203681 (PMC8610117; doi:10.18632/aging.203681)
Supplement: Supplementary Figures [file aging-13-203681-s001.pdf]

SUPPLEMENTARY FIGURES

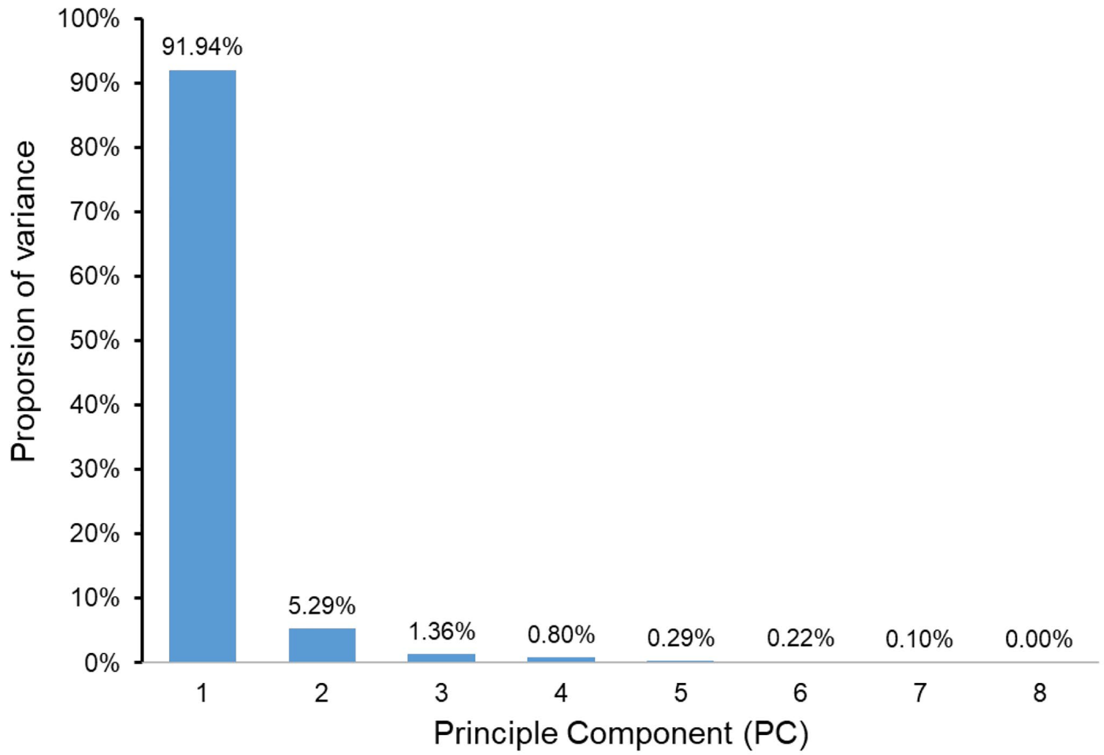

Supplementary Figure 1. Principal Component Analysis (PCA). Histogram of variance for each PC shows that the first PC captures the largest variance of dataset, which is 91.94%.

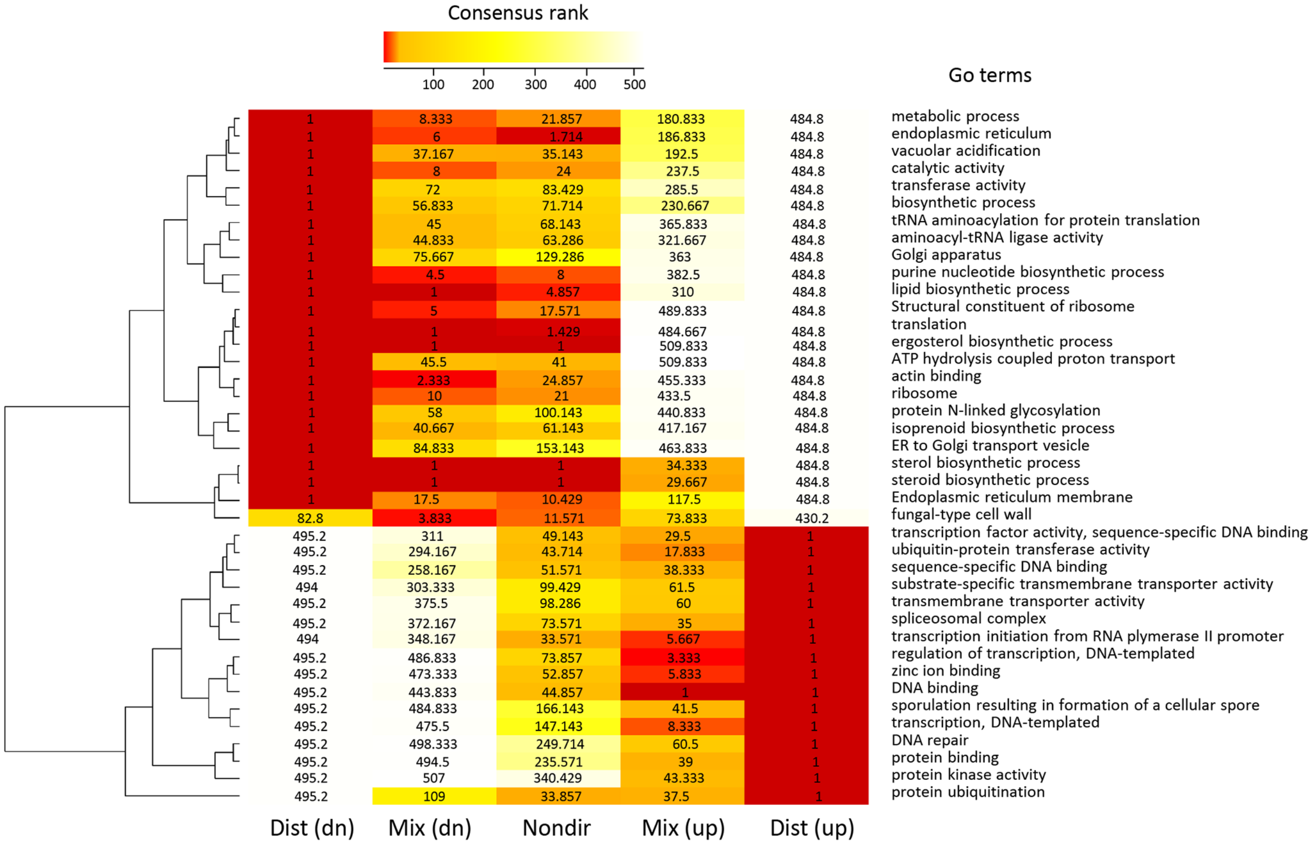

Supplementary Figure 2. The Heatmap of consensus scores of selected gene sets in L-UBB+1 strain comparing to control strain during EX phase. Consensus score is the mean rank given each gene set by different GSA runs. A low score (e.g., 1) is a gene set that is ranked high by most of GSA methods. Gene sets that received a median consensus rank <10 in at least one class from five classes (distinct-directional down, mixed-directional down, non-directional change, mix-directional up and distinct-directional up), are included in the heatmap. The ranking of gene set was shown by colors. Gene sets clustered at the upper part are showing patterns of mostly down-regulation whereas the gene sets in the lower part are showing patterns of mainly up-regulation. The scores are presented inside each cell of the heatmap.

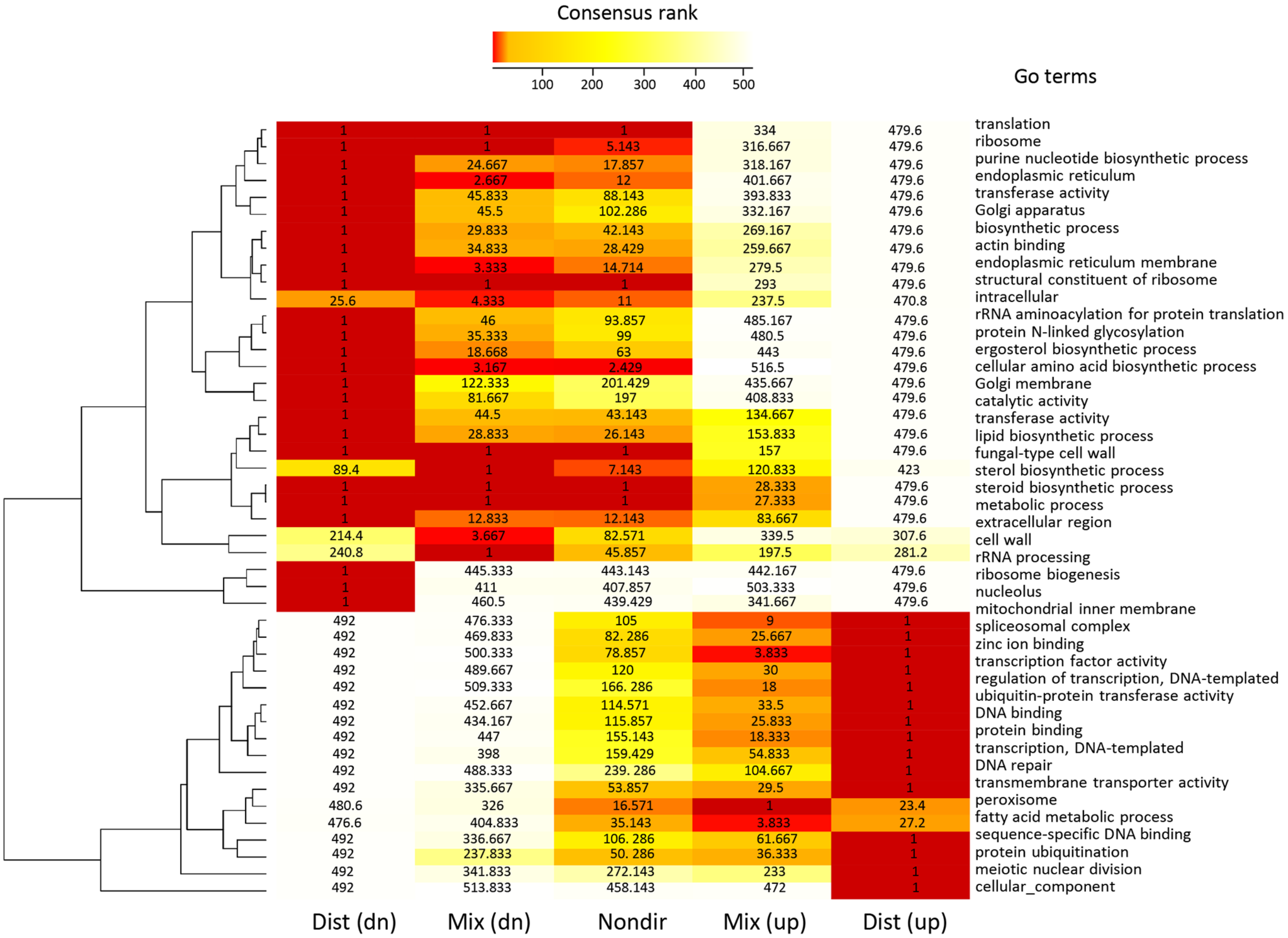

Supplementary Figure 3. The Heatmap of consensus scores of selected gene sets in L-UBB+1 strain comparing to control strain during D6 phase.

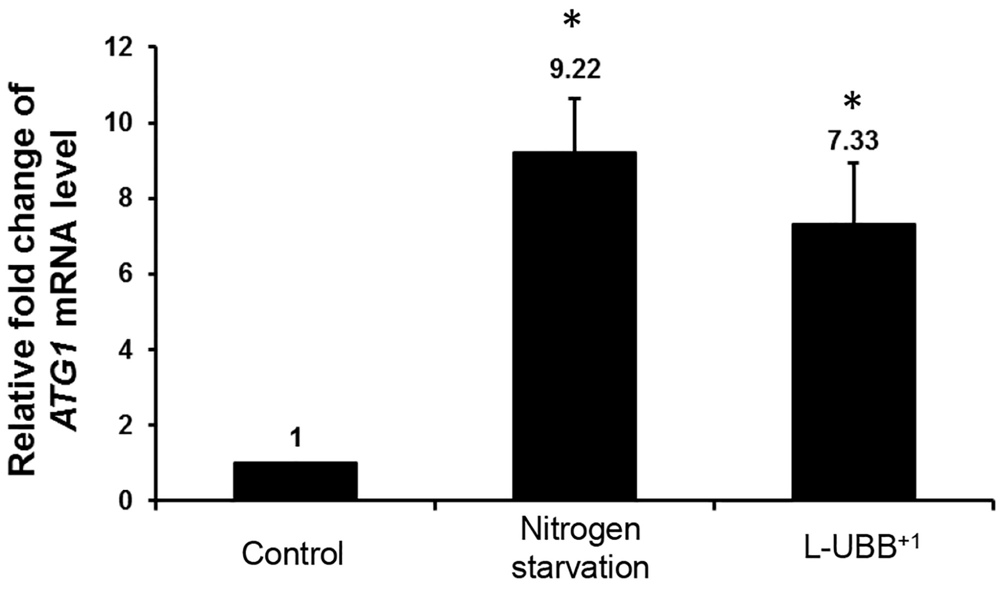

Supplementary Figure 4. qPCR analysis of *ATG1* mRNA expression in control strain and L-UBB+1 strain during EX phase. Nitrogen starvation was induced in YNB (-N) medium for 4 h after mid-EX phase. Results are normalized to *ACT1* mRNA level in control strain and shown as average values ± SD from biological triplicates. The asterisk (*) indicates significant difference compared to control strain ($p < 0.001$).

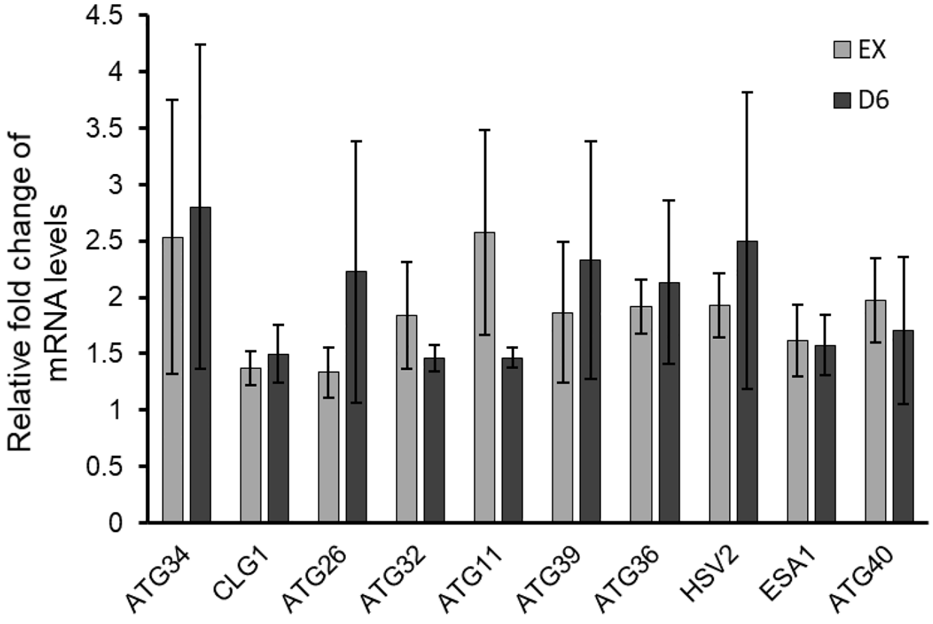

Supplementary Figure 5. The transcriptional response of autophagy related genes upon the L-UBB^{+1} expression during EX and PD phases. Results are normalized to *ACT1* mRNA level in control strain and shown as the average values ± SD from biological triplicates.

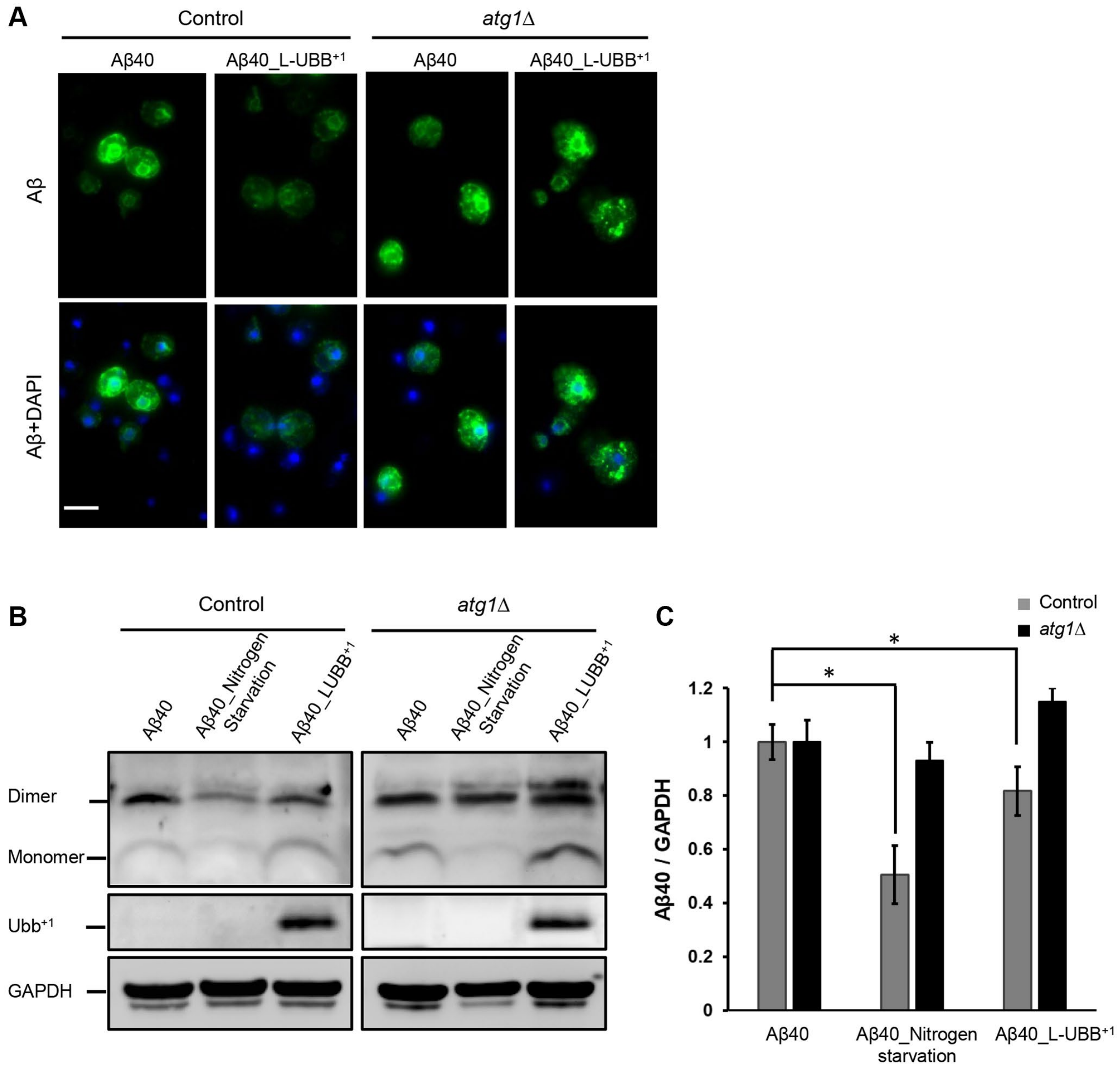

Supplementary Figure 6. Low UBB^{+1} expression reduces Aβ40 levels in the humanized yeast AD model. (**A**) Immunostaining analysis of Aβ40 localization and expression using the 6E10 Aβ specific antibody. Nuclei were stained blue by DAPI. Scale bar = 5 µm. (**B**) Western blot analysis of Aβ40 expression in unboiled cell lysates with 6E10 antibody. GAPDH was used as the loading control. (**C**) Relative Aβ40 band intensity was normalized to GAPDH and compared to the untreated Aβ40 strain. Results are shown as average value ± SD of three independent experiments. $^{*}p < 0.05$.
