# Supplementary material for: UBB+1 reduces amyloid-β cytotoxicity by activation of autophagy in yeast
Source: Aging (Albany NY). 2021 Nov 9;13(21):23953–80. doi: 10.18632/aging.203681 (PMC8610117; doi:10.18632/aging.203681)
Supplement: Supplementary Tables [file aging-13-203681-s002.pdf]

SUPPLEMENTARY TABLES

Supplementary Table 1. Overrepresentation of autophagy associated processes with up-regulated genes in L-UBB^{+1} strain.

Category description	No. of genes in dataset (EX/D6)	No. of genes in category	Fold enrichment (EX/D6)	*p*-value (EX/D6)
Mitophagy (GO:0000422)	20/23	48	2.05/2.30	$1.71 \times 10^{-3}/8.77 \times 10^{-5}$
Late nucleophagy (GO:0044805)	11/13	20	2.7/3.12	$2.91 \times 10^{-3}/1.52 \times 10^{-4}$
Nucleophagy (GO:0034727)	18/22	46	1.92/2.29	$6.69 \times 10^{-3}/1.36 \times 10^{-4}$
Regulation of autophagy (KEGG:sce04140)	8/11	17	2.76/3.63	$1.61 \times 10^{-2}/1.72 \times 10^{-4}$
Positive regulation of macroautophagy (GO:0016239)	7/8	12	2.87/3.20	$2.09 \times 10^{-2}/4.96 \times 10^{-3}$
Autophagy (GO:0006914)	29/38	100	1.43/1.82	$4.02 \times 10^{-2}/1.39 \times 10^{-4}$

p-value < 0.05 was used as a cutoff.

Supplementary Table 2. Genes with significantly different expression in autophagy pathways (L-UBB^{+1} strain vs control strain) adjusted *p*-value <0.05 was used to identify significance.

Systematic name	Standard name	fold change in EX	Adjusted *p*-value in EX	fold change in Day 6	Adjusted *p*-value in Day 6
YGL180W	ATG1	2.81	6.76E-06	2.56	9.56E-06
YLL042C	ATG10	0.68	2.03E-03	0.93	3.72E-04
YPR049C	ATG11	2.51	2.18E-06	2.29	2.62E-06
YBR217W	ATG12	1.28	7.76E-05	1.60	2.46E-05
YPR185W	ATG13	0.64	6.50E-04	0.84	1.42E-04
YCR068W	ATG15	1.55	7.40E-05	1.34	1.53E-04
YLR423C	ATG17	0.12	6.21E-01	0.66	2.30E-02
YFR021W	ATG18	−0.85	7.66E-04	−0.73	1.60E-03
YOL082W	ATG19	−2.65	6.84E-06	−1.63	6.21E-05
YNL242W	ATG2	0.84	2.74E-03	0.94	1.50E-03
YDL113C	ATG20	1.03	2.85E-04	1.49	3.84E-05
YLR431C	ATG23	1.06	2.41E-04	1.45	4.45E-05
YLR189C	ATG26	2.76	2.55E-06	2.86	2.20E-06
YJL178C	ATG27	−1.57	9.21E-05	−1.50	1.10E-04
YNR007C	ATG3	1.85	1.37E-05	2.32	5.42E-06
YDR022C	ATG31	0.73	3.32E-02	1.50	1.13E-03
YIL146C	ATG32	3.10	1.19E-06	3.26	8.45E-07
YLR356W	ATG33	0.13	7.64E-01	0.80	7.17E-02
YOL083W	ATG34	2.30	3.30E-04	3.06	6.93E-05
YJL185C	ATG36	1.46	9.96E-04	1.84	2.74E-04
YLR211C	ATG38	−0.50	3.27E-02	−0.30	1.60E-01
YLR312C	ATG39	4.15	1.36E-06	4.56	8.45E-07
YNL223W	ATG4	1.30	2.06E-05	1.49	1.10E-05
YOR152C	ATG40	2.06	1.06E-04	2.53	3.52E-05
YPL149W	ATG5	−1.00	4.08E-05	−0.70	2.65E-04
YPL120W	ATG6	0.15	3.26E-01	0.33	4.75E-02
YHR171W	ATG7	0.35	2.96E-01	0.75	4.67E-02
YBL078C	ATG8	2.14	6.55E-05	2.88	1.48E-05
YDL149W	ATG9	1.48	1.29E-04	2.09	2.14E-05

YJL095W	BCK1	0.42	3.54E-03	0.40	4.31E-03
YML077W	BET5	−1.20	1.84E-05	−1.04	3.49E-05
YDR022C	CIS1	0.73	3.32E-02	1.50	1.13E-03
YGL215W	CLG1	1.84	2.66E-05	1.38	1.08E-04
YBR109C	CMD1	−0.86	1.31E-02	−0.66	3.66E-02
YFL024C	EPL1	1.67	3.01E-05	1.32	9.73E-05
YOR244W	ESA1	2.03	6.59E-06	2.05	6.19E-06
YNL127W	FAR11	0.75	5.64E-04	0.64	1.35E-03
YGR163W	GTR2	0.19	1.23E-01	0.37	1.15E-02
YGR223C	HSV2	1.89	1.04E-05	2.21	5.53E-06
YPL250C	ICY2	−1.60	4.76E-04	−2.08	1.10E-04
YHR082C	KSP1	1.45	7.74E-06	1.37	9.32E-06
YKR007W	MEH1	0.72	3.39E-04	1.03	5.10E-05
YPL140C	MKK2	1.65	4.59E-06	1.69	3.99E-06
YGL124C	MON1	−0.60	9.36E-03	−0.58	1.07E-02
YMR004W	MVP1	0.68	3.33E-04	0.92	6.37E-05
YEL062W	NPR2	−0.55	6.86E-03	−0.78	1.17E-03
YHR195W	NVJ1	0.27	2.43E-01	0.66	1.60E-02
YNL289W	PCL1	−2.66	2.38E-05	−2.68	2.23E-05
YHR071W	PCL5	1.25	3.75E-03	1.11	6.53E-03
YPL154C	PEP4	−0.91	6.97E-03	−0.59	4.18E-02
YOL001W	PHO80	0.91	5.88E-05	1.04	2.82E-05
YPL031C	PHO85	−0.01	9.41E-01	0.33	3.20E-02
YDR435C	PPM1	0.52	3.15E-03	0.77	3.84E-04
YNL330C	RPD3	0.73	1.23E-03	0.69	1.60E-03
YBL103C	RTG3	0.66	6.03E-03	0.50	1.99E-02
YPL085W	SEC16	0.49	2.04E-03	0.46	2.72E-03
YBL050W	SEC17	1.08	4.36E-05	1.39	1.29E-05
YNL272C	SEC2	0.57	1.64E-03	0.59	1.42E-03
YFL005W	SEC4	0.28	4.12E-02	0.60	1.19E-03
YBL058W	SHP1	0.73	6.54E-04	0.93	1.72E-04
YLR079W	SIC1	0.70	5.67E-04	0.88	1.65E-04
YBR077C	SLM4	1.05	1.44E-04	1.39	3.25E-05
YJL036W	SNX4	1.41	1.89E-05	1.67	8.55E-06
YAR042W	SWH1	1.43	1.42E-04	1.40	1.49E-04
YJR066W	TOR1	0.55	2.66E-03	0.41	1.08E-02
YKL203C	TOR2	1.04	1.44E-04	0.86	3.77E-04
YKR042W	UTH1	0.84	9.09E-03	0.25	3.22E-01
YDL077C	VAM6	0.33	3.29E-02	0.35	2.71E-02
YOR043W	WHI2	0.64	1.00E-03	0.39	1.09E-02
YPL120W	VPS30	0.15	3.26E-01	0.33	4.75E-02
YLR396C	VPS33	−0.91	1.11E-04	−0.98	7.02E-05
YOL105C	WSC3	0.72	1.59E-03	0.37	3.11E-02
YFL004W	VTC2	0.55	8.50E-04	0.41	3.86E-03
YPL019C	VTC3	0.51	4.11E-02	0.61	2.08E-02
YLR312C	YLR312C	4.15	1.36E-06	4.56	8.45E-07
YOR019W	YOR019W	1.25	8.12E-04	1.40	4.32E-04
YOR152C	YOR152C	2.06	1.06E-04	2.53	3.52E-05

YFL038C	YPT1	0.44	3.33E-03	0.54	1.10E-03
YGL210W	YPT32	0.54	1.15E-03	0.71	2.60E-04
YML001W	YPT7	−1.18	6.56E-05	−0.87	3.15E-04
YHR030C	SLT2	−1.27	1.24E-05	−1.40	6.15E-06

Supplementary Table 3. Source data for Figures 5 and 7.

Figure 5A

Strain	Day 1	Day 3	Day 5	Day 7	Day 9	Day 11	Day 13	Day 15
control_1	343	306	329	249	75	24	13	24
control_2	306	345	369	244	46	21	10	10
LUBB+1-1	401	372	406	404	310	157	53	
LUBB+1-2	410	384	437	398	258	73	33	
atg1Δ_LUBB+1-1	453	365	327	237	77	19		
atg1Δ_LUBB+1-2	435	394	341	203	99	24		
atg1Δ_1	355	281	302	246	118	3		
atg1Δ_2	406	305	257	225	101	14		

Figure 7A

	Day 1	Day 3	Day 5	Day 7	Day 9	Day 11	Day 13	Day 15
control 1	343	306	329	249	75	24	13	
control 2	306	345	369	244	46	21	10	
Aβ42_1	393	313	94	39	7			
Aβ42_2	391	374	101	32	4			
Aβ40_1	458	348	278	110	40	7		
Aβ40_2	402	453	296	139	22	12		
Aβ42_LUBB+1_1	326	272	319	299	133	48	22	9
Aβ42_LUBB+1_2	290	268	266	214	132	42	29	10
Aβ40_LUBB+1_1	295	371	316	309	192	64	48	13
Aβ40_LUBB+1_2	315	347	389	290	156	67	25	23

Figure 7B

	Day 1	Day 3	Day 5	Day 7	Day 9	Day 11
atg1Δ_Control_1	355	281	302	246	118	3
atg1Δ_Control_2	406	305	257	225	101	14
atg1Δ_Aβ 42_1	441	451	241	5		
atg1Δ_Aβ 42_2	456	418	248	11		
atg1Δ_Aβ 40_1	476	504	165	2		
atg1Δ_Aβ 40_2	523	475	139	0		
atg1Δ_Aβ 40_LUBB+1_1	354	300	209	154	68	5
atg1Δ_Aβ 40_LUBB+1_2	334	263	235	183	55	8
atg1Δ_Aβ 42_LUBB+1_1	368	289	218	160	72	17
atg1Δ_Aβ 42_LUBB+1_2	364	242	196	136	53	5

Supplementary Table 4. Primer-sets used in this study.

Primer	Sequence (5′–3′)	Description
Atg1_up_fw_1<br>Atg1_up_rev	ACCGCTCGGCTCTGATTTCTTAAACC<br>CTGCAGCGTACGAAGCTTCAGTTTCTTAATTTCTCGTCTGGTG	Amplification of upstream of *ATG1* from genomic DNA
Atg1_down_fw<br>Atg1_down_rev	GTGATATCAGATCCACTAGTGGCCGTGATGCATAATATGGTTTTC<br>GGATCTAAGTTAATTGTCATGTCGG	Amplification of downstream of *ATG1* from genomic DNA
KanMX_fw<br>KanMX_rev_1	CACCAGACGAGAAATTAAGAAACTGAAGCTTCGTACGCTGCAG<br>GAAAACCATATTATGCATCACGGCCACTAGTGGATCTGATATCAC	Amplification of *KanMX* from pUG6 plasmid [83]
Atg1_up_fw_2<br>KanMX_rev_2	CGCATTACACAGCTGCTCCGGAC<br>TCACCATGAGTGACGACTGA	Verification of *KanMX* replacement of *ATG1*
Atg1_fw<br>Atg1_rev	TTGAGAACAGGCGCAGTATG<br>AAGGATCATTTCCGAACGTG	qPCR primers for *ATG1*
Actin_fw<br>Actin_rev	GCCTTCTACGTTTCCATCCA<br>GGCCAAATCGATTCTCAAAA	qPCR primers for *ACT1*
ATG39_fw<br>ATG39_rev	GAGGGGTCGAAACTGAAGGA<br>AAACCTGCCAACACATCACC	qPCR primers for *ATG39*
ATG32_fw<br>ATG32_rev	ACTGGGGAAGACAAAGGCTT<br>ATGAAAGAAGCGCCCAAGTC	qPCR primers for *ATG32*
ATG26_fw<br>ATG26_rev	AAATTCCGCTGCCCAACATT<br>TTTGACCTGACTACCGGACC	qPCR primers for *ATG26*
ATG11_fw<br>ATG11_rev	GCAGACGTAGATCTTTCGCG<br>TGTGAGCAAACGGTTAAGCC	qPCR primers for *ATG11*
ATG34_fw<br>ATG34_rev	ATGGGACCGCATGAGATAGG<br>GTGTGGAAACTGCCTGTCTG	qPCR primers for *ATG34*
ATG40_fw<br>ATG40_rev	AGACCCTTTGTAACGGAGCA<br>TCATTCGGGAACTCAGTGCT	qPCR primers for *ATG40*
ESA1_fw<br>ESA1_rev	CCGCGGATGGTTACAATGTT<br>AGCGTTATGAGAGTGTCCGA	qPCR primers for *ESA1*
HSV2_fw<br>HSV2_rev	GCAAACATCTCCAGTCGCAA<br>TGATGGAAGTGGGCAAAAGC	qPCR primers for *HSV2*
CLG1_fw<br>CLG1_rev	AAGGTTCGGCTACTTCTGCT<br>GGGAAGGATAGGTGGTTGCT	qPCR primers for *CLG1*
ATG36_fw<br>ATG36_rev	GGTGTTTGGGCAGCCATTTA<br>TGTCAATAATTGCCGGCGAG	qPCR primers for *ATG36*

Supplementary Table 5. Plasmids and *Saccharomyces cerevisiae* strains used in this study.

Name		References
Plasmid	Characteristics	
p413 TEF	CEN, *TEF1* promoter, *HIS3* marker	[84]
p413 TEF-UBB^{+1}	p413TEF with UBB^{+1} sequence	[27]
p416 GPD	CEN, *GPD1* promoter, *URA3* marker	[84]
p416 GPD-Kar2-Aβ42	p416GPD with Kar2 and Aβ42 sequences	[28]
p416 GPD-Kar2-Aβ40	p416GPD with Kar2 and Aβ40 sequences	[28]
pRS416 GFP-Atg8	pRS416 with GFP and Atg8 sequences	[85]
Strain	Relevant genotype	
CEN.PK 113-11C	*MATα his3Δ1 ura3-52 MAL2-8c SUC2*	[82]
Control	CEN.PK 113-11C/p413 TEF	
L-UBB^{+1}	CEN.PK 113-11C/p413 TEF-UBB^{+1}	
Aβ42	CEN.PK 113-11C/p416 GPD-Kar2-Aβ42	[28]
Aβ40	CEN.PK 113-11C/p416 GPD-Kar2-Aβ40	[28]

Aβ42_L-UBB^{+1}	CEN.PK 113-11C/p416 GPD-Kar2-Aβ42/p413 TEF-UBB^{+1}	This study
Aβ40_L-UBB^{+1}	CEN.PK 113-11C/p416 GPD-Kar2-Aβ40/p413 TEF-UBB^{+1}	This study
GFP_Atg8	CEN.PK 113-11C/p413 TEF / pRS416 GFP-Atg8	This study
L-UBB^{+1}_GFP_Atg8	CEN.PK 113-11C/p413 TEF-UBB^{+1}/pRS416 GFP-Atg8	This study
*atg1*Δ	CEN.PK 113-11C/*atg1*::loxP-KanMX4-loxP	This study
*atg1*Δ_Control	*atg1*Δ/p413 TEF	This study
*atg1*Δ_L-UBB^{+1}	*atg1*Δ/p413 TEF-UBB^{+1}	This study
*atg1*Δ_Aβ42	*atg1*Δ/p416 GPD-Kar2-Aβ42	This study
*atg1*Δ_Aβ40	*atg1*Δ/p416 GPD-Kar2-Aβ40	This study
*atg1*Δ_Aβ42_L-UBB^{+1}	*atg1*Δ/p416 GPD-Kar2-Aβ42/p413 TEF-UBB^{+1}	This study
*atg1*Δ_Aβ40_L-UBB^{+1}	*atg1*Δ/p416 GPD-Kar2-Aβ40/p413 TEF-UBB^{+1}	This study
*atg1*Δ_GFP_Atg8	*atg1*Δ/pRS416 GFP-Atg8/p413 TEF	This study
*atg1*Δ_GFP_Atg8_L-UBB^{+1}	*atg1*Δ/pRS416 GFP-Atg8/p413 TEF-UBB^{+1}	This study
